# Treatment seeking experiences of ACS patients in Yogyakarta, Indonesia during COVID-19 pandemic: A qualitative study

**DOI:** 10.1371/journal.pone.0302320

**Published:** 2024-04-30

**Authors:** Pramitha Esha Nirmala Dewi, Montaya Sunantiwat, Montarat Thavorncharoensap, Sitaporn Youngkong, Surakit Nathisuwan, Bangunawati Rahajeng

**Affiliations:** 1 Doctor of Philosophy Program in Social, Economic, and Administrative Pharmacy, Department of Pharmacy, Faculty of Pharmacy, Mahidol University, Bangkok, Thailand; 2 Department of Pharmacy Profession, Faculty of Medicine and Health Sciences, Universitas Muhammadiyah Yogyakarta, Bantul, Indonesia; 3 Social and Administrative Pharmacy Excellence Research (SAPER) Unit, Department of Pharmacy, Faculty of Pharmacy, Mahidol University, Bangkok, Thailand; 4 Health Technology Assessment Graduate Program, Mahidol University, Bangkok, Thailand; 5 Clinical Pharmacy Division, Department of Pharmacy, Faculty of Pharmacy, Mahidol University, Bangkok, Thailand; School of Health Binawan: Universitas Binawan, INDONESIA

## Abstract

Delay in treatment seeking is recognized as a major contributor for Acute Coronary Syndrome (ACS) mortality in Indonesia. Despite the significance of timely treatment, decline in admission and delay in presentation of patients with ACS were consistently reported during the COVID-19 pandemic. These suggested that treatment seeking performance of patients during the pandemic might be different from the previous period. This qualitative study aimed to explore treatment seeking behaviour, barriers in seeking medical treatment, and experiences of patients with ACS in Yogyakarta, Indonesia during COVID-19 pandemic. In depth-interviews were carried out with 30 patients, who were hospitalized with ACS at one of the three selected hospitals in Yogyakarta during the pandemic period. Thematic analysis was performed to create vital explanations for treatment seeking practices of patients with ACS during pandemic. Three significant themes were identified: treatment seeking decisions, barriers in seeking medical treatment during COVID-19, and experiencing both good and bad impression from entering and staying in the hospital. The intensity of ACS symptoms and fear of COVID-19 infection dominated the delay in seeking medical treatment. Strict safety measures, religious belief, and fear of ACS helped patients overcome barriers and seek medical treatment during pandemic. ACS patients also had convenient medical care during the pandemic and believed medical staff would provide excellent care to them. However, visit restriction policy could cause psychological discomfort. Increase awareness of ACS symptoms and the risk of delays ACS treatment are essential to support patients’ decisions to seek medical helps in a timely manner at any situations including pandemic. Interventions aim at alleviating psychological distress should also be designed and implemented to improve treatment experiences of ACS patients, who sought medical treatment during the pandemic crisis.

## Introduction

During the outbreak of COVID-19 pandemic healthcare services have been substantially disrupted leading to negative health consequences and excessive deaths. As of 9 August 2023, there have been 769.4 million confirmed cases of COVID-19, including 6.9 million death, globally [1]. Besides the devastating numbers of death from COVID-19 infection, the pandemic also contributed to the massive reduction in health service utilization, especially among patients with less severe conditions [2]. Notably, the reduction of essential treatment services may lead to complications and significant deaths.

Acute Coronary Syndrome (ACS) is a life-threatening heart condition that requires immediate treatments to prevent adverse cardiac events and mortality risk [3–5]. Prior to the pandemic, pre-hospital treatment delays, which is a major cause of mortality among patients with ACS, have been reported in many low-and middle-income countries [6]. Lack of awareness of signs and symptoms of ACS, lack of adequate Emergency Medical Services (EMS), limited access to transportation, and delay decision to seek treatment are among important factors contributing to such delays [6]. During the pandemic, significant reduction in the number of ACS admissions and the increase in delayed presentation of ACS patients were consistently observed. These suggested that treatment seeking behaviors of patients with ACS during the pandemic might differ from the prior period [7–9].

Indonesia is the fourth most populous country in the world, located in Southeast Asia. ACS is considered a major cause of mortality and disability in Indonesia [10]. In 2019, Indonesia was ranked as the 6th country in the world with the highest number of cardiovascular deaths [11]. Prior to the COVID-19 pandemic, unmet timely care among ACS patients has been a major concern in Indonesia [12–14]. Starting from 3 January 2020 to 16 August 2023 [1], approximately 6,813,000 confirmed cases of COVID-19 and 161,916 deaths were reported in Indonesia.

It should be noted that several additional factors could profoundly affect treatment seeking behaviour/decision during the pandemic. These might include the fear of contracting COVID-19 [15], lock down policies [16], and stay-home advices [17]. In addition, several additional strategies such as COVID-19 screening procedure, isolation process of infected patients, visitor restriction, the use of personal protective equipment, and surface and equipment cleaning/decontamination process were implemented in the hospitals to mitigate the transmission [18]. Overcrowding and scarce medical resources have been widely reported during the pandemic [19]. These aforementioned factors could affect treatment seeking behaviour as well as treatment experiences of the patients during the pandemic. There is an urgent need to comprehend the treatment seeking behaviour of patients, the barriers that may cause delayed presentation of acute coronary syndrome (ACS) patients at healthcare facilities, and the treatment experiences of ACS patients during the pandemic. However, the available evidence on these matters is extremely limited [20, 21]. This qualitative study, therefore, aimed to explore patients’ treatment seeking behaviour, barriers in seeking medical treatment, and treatment experiences of patients with ACS during the COVID-19 pandemic. Findings from this study could serve as an important lesson for healthcare staffs and policy makers to develop suitable and practical strategies to promote timely decision to seek treatment among patients with ACS and to improve the quality of ACS care process during the future crisis.

## Methods

### Study design

In-depth interview was conducted for this qualitative study among patients, who were hospitalized with ACS during the pandemic at the three participated hospitals in Yogyakarta, Indonesia. An in-depth interview enables participants to express their thoughts and emotions more thoroughly and is an attractive method when a detailed report on a person’s opinion, feeling, experiences, and behaviour is needed. [22]. The participated hospitals consisted of one secondary public hospital, one secondary private hospital, and one tertiary academic hospital located in Yogyakarta, Indonesia. This study was conducted and reported in accordance with the SRQR criteria for reporting qualitative research [23]. The details of SRQR according to this report can be seen in the supporting file (S1 File).

### Participants

Study participants were patients with ACS, hospitalized at the three selected hospitals. Participants who met the eligibility criteria were purposively selected through the collaboration with nurses and cardiologists, who were currently working at the participating hospitals. Those included the ones who 1) aged 18 years and above, 2) were hospitalized with ACS during the COVID-19 pandemic, 3) were discharged with ACS not longer than 1 year before the date of interview, and 4) were willing to share their deep experiences regarding frightening symptoms, their urgent ACS treatment seeking performances, and their treatment experience during the pandemic.

### Data collection

The recruitment process was conducted within March 24 –August 4, 2022. Purposive sampling technique with the aims to maximize the richness and depth of the information that can address the research question was adopted [24]. Specifically, ACS hospitalized patients in the selected 3 hospitals were purposively chosen by nurses and cardiologists according to the specified criteria. Eligible participants who agreed to participate in the study were contacted by the research team to schedule a convenient day and time for the interview. Face-to-face in-depth interviews were performed and audio-recorded by the first author during the study period. The interview was conducted in the private room during their waiting period for regular visit at the cardiology outpatient department. A series of open-ended questions were asked to capture experiences in seeking care and treatment as follow: 1) Could you explain your symptoms before hospital admission? 2) What made you decide to go to the hospital? 3) What were the barriers in seeking treatment at the hospital during COVID-19 pandemic? 4) Could you tell us your problems and concerns in making decision to seek medical treatment during COVID-19 pandemic? and 5) Could you share us your experiences in seeking treatment at the hospital during COVID-19 pandemic? All participants signed for written informed consents prior to their enrolment in the study. Each interview lasted for 45–60 minutes. Data collection was conducted until data saturation was achieved.

The study was conducted in accordance with the declaration of Helsinki and ethical approval was obtained from the Health Research Ethics Boards from Universitas Muhammadiyah Yogyakarta (041/EC-KEPK FKIK UMY/II/2022) and Universitas Gadjah Mada (KE/FK/0322/EC/2022).

### Data analysis

In this study, thematic analysis was selected to create the experiences of participants with ACS in searching for fast and optimal treatments. All interview recordings were transcribed verbatim. Every transcript was firstly scanned to reach the main idea of each interview. The transcript, then, was read in details and some interesting words or sentences were highlighted in order to form “the golden words” representing some particular issues that can answer research questions. Afterwards, crucial issues that were relevant were connected, organized, and categorized in order to create main themes and subthemes to explain all research questions. All researchers collaborated in the analytic process to ensure consistency in interpreting the statements and expressions provided by the patients.

## Results

In-depth interviews were conducted among 30 patients. Of these, 22 were males while 8 were females. The age of the patients ranged from 40 to 63 years old. All were married. Participants’ characteristics were shown in Table 1. The following three themes were identified: 1) treatment seeking decision, 2) barriers in seeking medical treatment during COVID-19, and 3) experiencing both good and bad impression from entering and staying in the hospital. These main themes and eleven sub‐themes within them were discussed below along with the relevant quotations.

**Table 1:** Participant’s characteristics.

No	Participant ID	Age	Gender	Marital Status	Socio-economic status	Type of Hospital	Time of Interview After Discharge
1	MWO	60	Male	Married	Farmer	Tertiary	4 months
2	WSO	57	Male	Married	Farmer	Tertiary	6 months
3	NNG	50	Female	Married	Teacher	Tertiary	6 months
4	WRO	59	Male	Married	Entrepreneur	Tertiary	10 months
5	JKO	55	Male	Married	Entrepreneur	Tertiary	8 months
6	KSI	50	Male	Married	Engineer	Tertiary	4 months
7	EFI	63	Male	Married	Retired	Tertiary	5 months
8	AGS	45	Male	Married	Driver	Tertiary	1 month
9	FZY	47	Female	Married	Accountant	Tertiary	9 months
10	HDI	47	Male	Married	COVID-19 taskforce	Tertiary	7 months
11	DTO	48	Male	Married	Entrepreneur	Tertiary	4 months
12	HMH	55	Female	Married	Housewife	Secondary-Public	8 months
13	MLY	59	Male	Married	Farmer	Secondary-Public	6 months
14	WKJ	47	Male	Married	Builder	Secondary-Public	4 months
15	LGM	45	Male	Married	Farmer	Secondary-Public	4 months
16	PWR	60	Male	Married	Retired	Secondary-Public	3 months
17	RMN	60	Female	Married	Housewife	Secondary-Public	7 months
18	SYD	40	Male	Married	Gardener	Secondary-Public	9 months
19	SRT	47	Female	Married	Farmer	Secondary-Public	6 months
20	MYT	47	Female	Married	Housewife	Secondary-Public	4 months
21	PTO	48	Male	Married	Entrepreneur	Secondary-Public	10 months
22	SKO	45	Male	Married	Civil Servant	Secondary-Public	10 months
23	SDS	55	Male	Married	Farmer	Secondary-Public	5 months
24	STK	63	Male	Married	Retired	Secondary-Private	4 months
25	SFT	55	Female	Married	Entrepreneur	Secondary-Private	4 months
26	HYT	47	Male	Married	Migrant worker	Secondary-Private	11 months
27	PDO	45	Male	Married	Migrant worker	Secondary-Private	7 months
28	RKM	55	Male	Married	National Armed Force	Secondary-Private	10 months
29	SFR	47	Male	Married	Entrepreneur	Secondary-Private	9 months
30	SYM	63	Female	Married	Entrepreneur	Secondary-Private	6 months

### Theme 1: Treatment seeking decision

#### Subtheme 1.1 ‐ The more threatening expressions of the symptoms were perceived, the more immediate decisions for seeking treatments were driven

The feeling of sudden and excruciating pain along with other intolerable symptoms of ACS can drive both participants and their families to immediately seek effective medical treatment for their illness crisis at the hospital:

“Because of the excruciating pain. The priority was to get immediate treatment.” (LGM)

“I felt pain and heat in my chest, shortness of breath, and cold sweat appeared. Then, my wife immediately took me to the ER.” (PDO)

“Because of this terrible chest pain, I didn’t think about anything. The main goal was to go to the hospital immediately to get medicine.” (DTO)

#### Subtheme 1.2 ‐ Delay in seeking medical help owing to mild to moderate symptoms at the early phase

On the contrary, ACS can firstly be perceived as mild to moderate symptoms, such as tiredness and fatigue. These symptoms could be misinterpreted as common conditions resulted from hard work and lack of rest, or symptoms of common cold. Hence, participants decided to take some rest and used herbal or traditional remedies to easily alleviate their symptoms instead of seeking urgent medical treatment at the hospital. Nevertheless, the repeated episodes of pain and worsening symptoms ultimately triggered them to urge for effective seek medical treatment at the hospital.

“At home, I rubbed and massaged my arms and chest with oil because I thought that I caught a cold. But I did not feel better.” (MYT)

“I thought I was just tired. Then an hour later, it hurt again until there was a lot of cold sweat. My wife, then, rubbed my back with some oil but it didn’t improve.” (SYD)

“At first, I didn’t want to go to the hospital in the morning because I thought I would get better. Later, it got worse. So, I was taken to the closest hospital in afternoon.” (WSO)

“The chest pain was getting more and more frequent. So yes, I guess I really had to go to the hospital.” (HYT)

#### Subtheme 1.3 ‐ I and my significant person involved in treatment seeking decision

Some patients made their own decisions to search for medical treatment. These occurred among patients who had good knowledge on signs and symptoms of ACS as well as the advantages and importance of immediate medical treatment. This knowledge could trigger them to immediately seek urgent medical help at the hospital. Furthermore, patients’ expectation for recovering and maintaining healthy conditions triggered them to immediately seek medical health at the hospital.

“Because I need medicine to treat my heart condition. If I didn’t come to the hospital, I was sure my heart would relapse again. That was what I was afraid of” (RKM).

“My concern was only related to my heart condition. At that time, my condition required immediate medical help.” (PDO)

“The only concern at that time was to get help as soon as possible. Furthermore, I wanted to be recovered, healthy, and not experience severe pain like that anymore” (SFR).

For some patients, the severe symptoms could shut down their abilities to make decisions for efficient treatment. As a result, family members made decision on behalf of the patients or persuaded patients to seek medical treatment:

“I did not have a choice at that time. It was my wife and son who made their decision to take me to this hospital.” (WRO)

“This happened again and again for about four days. On the 4th day, wow, I couldn’t stand it anymore. My son decided to take me to the hospital.” (MWO)

“If my daughter-in-law didn’t convince me to get treated, I wouldn’t have come here for treatment.” (SYM)

Moreover, neighbours could also play an important role to support patients to urge for instant medical treatment at the hospitals. With the help of neighbours, lives of the ACS patients can be saved from delay in seeking medical help:

“At that time, my neighbour took me to the hospital directly.” (SYD)

“It was very early in the morning, I felt like my chest was being stabbed with needles. Then, my mother and nephew took me straight away to this hospital using our neighbour’s car.” (LGM)

#### Subtheme 1.4 –Seeking medical rescue at the nearest hospitals

When encountered the unexpectedly serious symptoms, many participants and their families decided to seek the urgent medical help at the closest health care facilities:

“That afternoon, I was taken to the hospital near my house and was immediately admitted to the ICU.” (WSO)

“Then, I was immediately brought by my son on a motorbike with three people on it where, I was in the middle. At that time, I came here because it was the closest hospital to my house.” (MLY)

In addition, health insurance was considered as an important gate-keeper influencing the options for medical treatment seeking decision. According to the Badan Penyelenggara Jaminan Sosial (BPJS) insurance system, patients are required to receive their treatment packages only at their registered hospitals. If the patient’s condition are serious and cannot be solved by the staff in the hospital. They will be referred to the higher-level hospitals that staff are experts and medical instruments are prompt to provide the fast and effective treatments for the patients.

“As BPJS health insurance was free, I wanted to find out what my health problem was. That’s why I decided to go to the hospital.” (AGS)

“The process of going to this hospital was based on a referral from the previous hospital, which was complied with the regulation of BPJS.” (HDI)

“Then, I was referred to this hospital by BPJS insurance.” (DTO)

#### Subtheme 1.5 ‐ Despite the severe condition of COVID-19, my critical heart condition is much more threatening

When comparing between COVID-19 pandemic and seriousness of ACS, alleviation and recovery from critical ACS problem could overcome the fear of COVID-19 infection leading to immediate treatment. This can be vividly presented by some marginalized patients expressed their desperate desire to achieve fast recovery so that they could get back for their work as soon as possible:

“It’s clear that my problem is in the heart, so I had to find medicine for my heart at the hospital. Why worried about COVID, the problem was clearly in the heart, not COVID. The most important thing is following the safety protocol.” (SKO)

“I was from a poor family. So, whether or not there was COVID I still had to earn a living for the family. I trusted in God. I did everything to make sure that I could recover and be able to take care of my family. (DTO)

### Theme 2: Barriers in seeking medical treatment during COVID-19 pandemic

#### Subtheme 2.1 ‐ Fear of catching COVID-19 in the hospitals

During COVID-19 pandemic, hospitals were perceived negatively as the “risky sites” for spreading COVID-19 infection. Fear of being infected by COVID-19 virus was undeniably expressed by many ACS patients. The spreading news about the increasing number of COVID-19 cases and deaths was perceived as a horrible situation by the participants, especially among those with co-morbidities. These aforementioned situations trigger fearful feelings and make patients feel reluctant to approach and seek medical care at the hospital.

“The news kept reporting the number of patients, who died from COVID. My God! At that time, I thought it was really scary.” (STK)

In addition, the deaths due to COVID-19 infection were surrounded in participants’ lives. This seriously aggravated their fear more and more, especially when they had to be hospitalized:

“During the time I had a heart attack, it was reported that many of my neighbours died from COVID. Oh my God, I couldn’t sleep in the hospital, I was wondering if I might develop serious disease like that too. I was very afraid at that time.” (PWR)

#### Subtheme 2.2 ‐ The very long waiting time/que caused by the number of COVID-19 cases

Even though being accepted to stay in the hospital, amid participants’ anxiety and fear of catching COVID-19, the number of patients in the waiting area was considered as another barrier causing most participant feel reluctant to seek and receive medical treatment.

“In my opinion, a very long queues at the outpatient polyclinic during the pandemic made it more inconvenience.” (PDO)

“Before I got COVID-19, I felt uneasy and scared every time I watch the news. It was reported that so many patients could not get a hospital room and that their beds were put in the hallways.” (SKT)

### Theme 3: Experiencing both good and bad impression from entering and staying in the hospital

Despite the chaotic situation of COVID-19, participant’s satisfaction was vividly expressed. Patient’s impression was also explored during their visit in the hospital at pandemic era.

#### Subtheme 3.1 ‐ Sudden and severe conditions were promptly and effectively handled as soon as arriving at the emergency room

The rapid process in handling patients during their emergency visits was impressed and recognized by the most participants:

“I felt that the doctors and nurses in the ER were quite fast in handling my condition.” (PDO).

“When I arrived here, I went straight to the ER. I was immediately examined and swabbed for COVID infection. The doctor was quite fast and I felt better not so long after.” (HMH)

“At that time the process was very fast. I arrived here before 6 am, then around 7 am I was already admitted to the ICCU for further treatment.” (LGM)

In addition, ability of medical staff in communicating and explaining about medical diagnosis and procedures to the participants and their families were also effective contributing to treatment satisfaction among most of the participants:

“Thank God! The doctor clearly explained to me why I was sick and what I needed to recover. He also explained why I had to move to another hospital. It was all well-explained. So, my family and I were not confused.” (SDS)

#### Subtheme 3.2 ‐ Feeling safe and relaxed by the standardized safety protocol and believing that everything is under control by God’s power

Reassured by the standard guidance and regulations to prevent COVID-19 infection provided by the government and clarify informed in the hospitals, most participants felt secured and relieved that their lives would be safe during hospitalization. Feelings grave concerns and fear from being infected by COVID-19 were alleviated. Furthermore, keeping the faith in God’s will, they perceived that everything, including their operations, would be under control and run smoothly with the power of God.

“It’s important for me to wear mask and wash my hands. I’m not afraid, I trust everything in God” (SFT).

“I do believe that health and illness are completely destined by God. We, as humans, can only follow the rules/regulation, and the way of life. No need to be afraid to overthink, trust everything in God” (HYT).

“As long as we followed government advice everything would be fine. My doctor also provided assurance to me.” (NNG)

#### Subtheme 3.3 ‐ Inconveniences and communication difficulties during hospital visits/hospitalization were reported

Some participants expressed the negative impacts of visiting restriction during the pandemic. Nevertheless, an online application could be implemented to alleviate such problem.

“When I was in the isolation room, after the second attack, I asked the nurse to contact my family. So, I could video call my family while I was there. Thank God! It’s allowed and I felt more comfortable.” (HMH)

“It was inconvenient to be hospitalized during COVID. Only one family member was allowed to stay with patient. This family member had to undergo a swab test. In my case, if my wife was tired and wanted to be replaced by other family members, it was difficult. It made me sad as only my wife had to accompany me for the whole week in the hospital.” (LGM)

Some participants mentioned that protective measures such as masks, gown, and social distancing hindered the communication with healthcare staffs:

“It was hard to communicate when the doctor was covered by many layers of clothing. During the examination procedure, when everything was covered, it was hard to communicate. I could not hear what the doctor said.” (SFT)

However, most of the participants expressed that under the thick cover of mask and suit, all medical professionals tried to explain the standard protocol and procedures clearly and slowly to make sure that their patients can understand the key contents that they would like to emphasize. This made most participants felt very thankful for their attempt and kindness.

#### Subtheme 3.4 –Under the strict and rigid regime of hospital visit, participants felt safer and a lot more secured

A strict measures and regulations of the hospital along with the presence of healthcare workers made participants felt safe and secured during the hospital visits or hospitalization:

“I was confident because the procedures were strict and there were a lot of health workers there.” (AGS)

“Even if I got COVID I was already in the hospital anyway. There would be many doctors treating me, so I was not worried.” (LGM).

## Discussions

This study provided insight into treatment seeking behaviour, barriers and enabling factors for seeking medical treatment, and experiences after entering hospitals, communicating with healthcare staffs, and receiving medical treatment for ACS patients during the COVID-19 pandemic. We found that treatment seeking decision was aligned with the evidences prior to the pandemic in that mild ACS symptoms led to the delay in seeking medical treatment [25–28]. When symptoms were mild, such as fatigue and tiredness, patients might misinterpret and believed that these symptoms were caused by hard work and could be fully recovered in a few days without specific medical treatment. Therefore, many participants with mild symptoms preferred treating themselves with traditional/herbal medicine to seeking immediate medical treatment. Like previous study in Indonesia [29], our study found that many participants with ACS relieved their symptoms by only massaging and rubbing their arms or chest with oil as applied in the medical pluralism concept [30].

Similarly, tendency to choose self-treatment by using home remedies to solve their symptoms was also found by previous study among women in Middle East country [25]. A previous review found that low intensity symptoms were considered as not serious disease then leading patients to delay for seeking medical helps [26] or just wait and believe that the symptoms will be away in the near future [28]. However, when symptoms emerged again and again, or when symptoms were severely aggravated, patients were triggered to seek effective medical care.

Fear of being afflicted with COVID became the main obstacle for patients to seek medical help in the healthcare facilities. In our study, fear of approaching and staying in the hospital was driven by media and numbers of dead patients in the hospitals announced every day. These have been reported in other studies [20, 31, 32]. However, for some patients, fear of severe ACS condition overcame that of COVID-19 infection. Thus, leading them to urge for medical treatment.

Our study also found that confidence in safety measures proposed and recommended by the Indonesian government were frequently and strongly expressed by most participants. A prior investigation concerning the relationship between trust in the government and behavioural reactions amidst the COVID-19 pandemic revealed that patients have faith in power of their government in managing or controlling health care system and spaces to achieve the safe and hygienic zone of all patients. Hence, they were willing to enter the hospital with confidence. [33]. Thus, our findings indicate that a segment of Indonesian society has a high level of faith in the government and health system while dealing with the pandemic.

In our study, some participants mentioned that their concern and fear were considerably alleviated and resolved by the power of God. This is consistent with the Muslim’s belief that health is one of the greatest blessings from God while illness is seen as part of the human condition by God’s wills [34]. Therefore, they tended to put all their faith in God and feel less fear. In summary, the health seeking behaviours identified from our study findings were in line with the concept of health belief model [35] in that health seeking behaviour was influenced by the following factors; 1) Perceived susceptibility to the disease, 2) Perceived severity of the disease, 3) Perceived benefits of the intervention, 4) Perceived barriers,, and 5) cues for action. According to our findings, patients who perceived that he/she might be susceptibility to ACS and those who experienced severe symptoms were likely to seek immediate treatment. On the other hands, fear of COVID-19, and perceived of overcrowding environment in the hospitals were identified as barriers for treatment seeking behaviour. However, when the symptoms were more severe so that the perceived threat of the ACS outweigh the fears of COVID-19 together with the perception on the benefit of medical treatment, the decision to seek medical treatment was, then, made. Moreover, the advices from others such as family members and neighbours, and advices from government especially about health care hygiene for preventing COVID-19 were served as the cue to action identified in our study.

Based on these findings, media campaign should focus on the value of the immediate treatment for ACS patients by emphasizing these specified symptoms as “severe” and “must go to the hospital”, as well as benefits of timely treatment to encourage patient’s decision to seek ACS treatment in the midst of pandemic. Fear of COVID-19 contracting, which was the main barrier for seeking medical treatment should also be minimized by disseminating the information that the effective prevention strategies were implemented at the hospitals to limit the transmission during the pandemic.

While other studies found that living alone [36] or reluctant to bother other [6] were cited as barriers for treatment seeking for ACS it is not the cases in our study. Similar to the pre-COVID period, we found that the treatment-seeking decision involved discussion with family members and neighbours. Involvement of family members in treatment decision-making is rooted in family and culture of Indonesia. Culturally, for both in rural and urban area, family plays an important role to help the patients in treatment decision-making process [37, 38]. In addition, the involvement of neighbours is also common in Indonesia [38]. According to Javanese culture, members of the community have a strong need to be in close proximity with one another and seem to have a harmonious and tight relationship with others in the society [39, 40].

In terms of treatment experiences, despite the fear of contracting COVID-19 prior to the hospital visits, patients were satisfied with medical services they received during the pandemic. In addition, they felt confident and safe while staying in the hospital and waiting for their surgery. This might be due to the fact that their safe health conditions were reassured by the very clean medical facilities and the very hygienic medical procedures, as it similarly reported by the previous study [21]. Moreover, all medical staff wore protective equipment and strict routine process to separate normal patients from those with COVID-19 were enforced continuously. Nevertheless, in order to maintain safe and secured health conditions for everyone, especially all vulnerable patients, duration to visit and talk with patient must be limited, numbers of family members allowed to meet each patient had to be restricted, and close contact between patients and guests was not permitted. As a result, most participants revealed that all restrictions during the pandemic made both patients and their lack of emotional support and cause much psychological distress. These negative impacts were vividly observed and experienced in various settings [20] and might have more severe effects on paediatric patients and patients with terminal illnesses, whose family support is extremely needed [41]. In fact, medical pluralism concerns of visiting restriction might affect patients’ decision to urge for medical treatment during the pandemic. Fortunately, with the advancement in technologies, several strategies including online applications, that could provide communication support between patients and their families were available and easily used. These technologies should be adopted for healthcare situation to improve emotional support and alleviate patient’s sufferings during the future crisis, especially when inpatient visit restriction has been seriously implemented.

Furthermore, negative consequences from protective medical devices such as surgical-mask and PPE clothes were identified as the barriers in communication. Specifically, masks and PPE clothes made it difficult to communicate with the health care providers. To improve the communication during the pandemic while still maintaining all standard protocols to ensure both patient’s safety and satisfaction, several strategies could be implemented and evaluated. These included increasing volume, articulating speech, emphasized on importance of non-verbal communication [42].

It is important to acknowledge that this study has certain limitations. Since half of the participants came from the moderate-to-low socioeconomic status, when perceiving symptoms like fatigue and tiredness, all were inclined to interpret these symptoms as “results from hard work” that was very common and normal in their everyday lives, not abnormal symptoms signifying the serious health conditions. Thus, they tended to neglect or tolerate to these trivial symptoms and still went for their work as usual instead of going for a medical check-up in the hospital. Hence, the result from our study cannot totally apply for those who were in the different socio-economic status. In addition, our study was conducted in Yogyakarta, a city rich in conservative culture, where relationship between family members and neighbours were still closed and tight. When one was in abnormal health condition, families, relatives, or neighbours were prompt and willing to help and accompany him/her to the hospital. If they considered that participant’s symptoms were serious, these relatives and neighbours encouraged or took participants to go to the hospital abruptly. Therefore, these findings could not be applied to the big and commercialized city, where relationships among people, including family and neighbours, were very loose and distanced. Furthermore, all hospitals selected in this study were very ready and effective in dealing with critical cases and also had strict and standardized procedures to control the pandemic situation very well. These cannot be generalized to all hospitals in Indonesia since ranges of standardized and hygienic protocol in dealing with both COVID-19 and other critical health conditions were various. Experiences of patients with ACS after entering the emergency room and staying in the hospital were different.

There are a number of potential biases that could influence the in-depth interview study. The initial source of bias pertained to the interviewer. The interviewer in this study (PEN) completed a one-semester course on qualitative research in order to prevent interviewer bias and was appropriately trained in interviewing techniques such active listening, open questioning and probing, and rapport-building to foster a rich information. The transcriptions were also examined by the team members in order to determine any situations in which the interviewer might have introduced bias into the interviewee’s response.

Secondly, we may be concerned about participant bias, which occurs when participants present themselves or their experiences in a manner that they perceive to be consistent with the expectations of the researchers. As the interviews were conducted in the hospital, some participants might feel reluctant to express some concerns on their treatment experiences. To mitigate the participant bias, our interviewer was trained to establish trust, and create a non-judgmental environment that encouraged open and honest sharing. Also, the interviews took place in a private room, during which the interviewer duly informed each participant that she was not affiliated with the hospital personnel and that any information provided would be treated as strictly confidential.

Thirdly, we recognized the potential for selection bias to manifest in our research. It should be noted that we relied on the nurses and cardiologists, whose comparatively greater familiarity with patient characteristics, to select our respondents. As a result, there might be the cases that we might missed some patients who had negative experiences on the ACS treatment. Furthermore, non-response bias could also be occurred. Unfortunately, we were unable to compare the characteristics of non-respondents and respondents. Lastly, recall biases could possibly occurred as participants were required to recall their behaviours and experiences during the hospitalization with ACS.

While many studies were conducted to examine the impact of COVID-19 pandemic on the delay in treatment seeking, clinical management, and outcome of patients with ACS [7, 8], very limited qualitative studies were conducted to gain more in-depth details in terms of treatment seeking behaviour and treatment experiences of patients with ACS during the pandemic [20]. Thus, our study provided useful information which reflected how patients seek treatment for this acute and life-threatening condition as well as their experiences during the crisis. Nevertheless, further studies examining experiences, barriers as well as strategies adopted to overcome ACS treatment provision among health care providers during the pandemic are also warranted.

## Conclusions

In terms of treatment seeking behavior, the lack of ACS symptoms recognition, especially the mild one, was considered a key non-COVID related factor contributing to delayed in seeking medical treatment. Fear of being infected is a significant barrier that hindered patients to seek medical treatment in the midst of pandemic. Family members and neighbours were the significant ones to persuade and carried the participants with critical symptoms of ACS to go for immediate treatment at the hospitals. The patient’s faith in God bolstered their motivation to seek medical assistance and undergo procedures to alleviate the symptoms of ACS.

With respect to treatment experience, most participants reflected their very positive impression from the fast and effective medical management as soon as entering the emergency room. In addition, they all experienced the hygienic and standardized procedures while staying in the hospitals and receiving surgery care and felt safe and relaxed. Nevertheless, under the very strict regime of serious disease control, opportunities for participants to communicate with medical staffs, their families and friends were inconvenient and difficult. Moreover, they mentioned positively that although all medical professionals were fully covered with surgical mask and tight costumes, they tried so hard to explain about details of participant’s conditions and medical treatment. Thus, they felt very satisfied and impressive with the attempt and kindness of all medical staffs.

Our study highlights the need of educational media and campaigns to promote the awareness of symptoms of ACS and the risk of delayed ACS treatment pervasively. During the future pandemic event, safety measures adopted by the hospitals and government to minimize risk of patients should be outline clearly to alleviate the fear of visiting hospitals and reassure that hospital is the safe place to seek immediate care for ACS. Innovative strategies, especially those involves technological advancement should be considered to mitigate the psychological distress from visit restriction policy.

## Supporting information

S1 File Standards for reporting qualitative research (SRQR). (PDF)

S2 File PLOS’ questionnaire on inclusivity in global research. (DOCX)
